# Suture length to wound length ratio for simple continuous abdominal closures in veterinary surgery: An experimental in vitro study

**DOI:** 10.1371/journal.pone.0215641

**Published:** 2019-04-26

**Authors:** Moriz E. Klonner, Brigitte Degasperi, Barbara Bockstahler, Gilles Dupré

**Affiliations:** Clinical Unit of Small Animal Surgery, Department for Small Animals and Horses, University of Veterinary Medicine Vienna, Vienna, Austria; University of Pisa, ITALY

## Abstract

**Objective:**

This study aimed to investigate the suture length to wound length ratio (SL:WL) in an in vitro model of abdominal wall closure. Effects of the surgeon’s experience level on the SL:WL ratio were evaluated, hypothesizing that small animal surgeons do not spontaneously apply SL:WL ratios equal to or larger than 4:1.

**Procedures:**

Three groups of surgeons with varying levels of experience performed 4 simple continuous sutures before (3 sutures) and after (1 suture) being educated on principles of the SL:WL ratio. All sutures were evaluated for their gaping, number of stitches, stitch intervals, tissue bite size and suture length.

**Results:**

No significant differences in suture parameters or SL:WL ratios were found among the 3 groups, and 60.5% of control sutures and 77.0% of test sutures had SL:WL ratios above 4:1. There was a significant improvement in the mean ratio after the information was provided (p = 0.003). Overall, the SL:WL ratios ranged from 1.54:1 to 6.81:1, with 36.3% falling between 4:1 and 5:1 (5.17 mm mean stitch interval, 5.52 mm mean tissue bite size). A significant negative correlation was observed between the SL:WL ratio and the stitch interval to tissue bite ratio (r = -0.886). Forty-nine of 120 sutures fulfilled the current recommendations for abdominal wall closure with a mean SL:WL ratio of 4.1:1.

**Conclusion:**

A SL:WL ratio larger than 4:1 was achieved in 60% of the control sutures and in 77% of test sutures. Additional animal studies are necessary to evaluate the SL/WL ratio in small animal surgery.

## Introduction

Incisional hernias are a frequent and unpredictable complication after midline laparotomy surgeries on humans; depending on the definition, their incidence rate in humans ranges from 4–23%.[1] The most common causes of incisional hernias are improper suturing techniques and wound infection, but suture breakage, knot slippage and untying are also possible causes.[2–4]

During the early period post-surgery, the closed wound relies on mechanical support until mechanically stable scar tissue is formed and is held together by only suture.[5, 6] Hypoproteinemia, metabolic diseases (such as diabetes mellitus and hyperadrenocorticism), immunosuppression, increased intraabdominal pressure and corticosteroid treatment may also increase the risk for dehiscence.[2–4] While infrequent, complications related to incisional hernias are often serious and costly.

Because incisional hernias are a frequent complication after midline surgical laparotomies in humans, many studies have focused on minimizing their risk of occurrence.[1] In 1976, Jenkins first introduced the theory of the suture length to wound length ratio (SL:WL), as he discovered that laparotomy wounds are in a constant dynamic state. After abdominal surgery, fascial layers may lengthen by 30% as the abdomen distends, causing a rise in suture tension. Furthermore, the ratio of suture used to the coeliotomy wound length is related to the incidence of incisional hernias.[7] Jenkins stated that deep wound disruption is associated with SL:WL ratios of 2:1 or lower and that wound disruption can be prevented by applying a SL:WL ratio of 4:1 or higher, as a higher ratio indicates that the tension increases to a lesser extent as the wound stretches. Jenkins supported this theory in both clinical trials and a mathematical approach.[7] Since Jenkin’s first approach, the SL:WL ratio has been the subject of many studies, and the risk of incisional hernia was shown to be more than three times higher when the abdomen is closed with an SL:WL ratio below 4:1.[8–10]

A high SL:WL ratio has a positive effect on not only the incidence of incisional hernias but also on the ultrastructural composition of the regenerative tissue.[5] Furthermore, the risk of wound infection with a ratio above 4:1 was two times lower than with a ratio below this threshold.[11] Finally, many studies have demonstrated that the SL:WL ratio can serve as an optimal foundation for the standardization of laparotomy closure techniques and is a perfect tool to monitor the quality of a surgeon’s suturing routine.[7, 9–12]

The SL:WL ratio depends on 3 parameters: suture tension, stitch interval and tissue bite size. Even though suture tension influences the SL:WL ratio, sutures that are too tight result in tissue ischemia, and those that are too loose result in wound gaping.

Höer presumed a widespread tendency among surgeons to apply a high suture tension,[13] which not only reduces tissue perfusion in the incisional area by up to 70% but also increases the risk of infection and the amount of pain in the healing phase.[14, 15] High suture tension leads to significantly lower collagen protein concentrations, causing the regenerative tissue to be significantly weaker.[16] Low suture tension, on the other hand, is less problematic, as it causes collagen I to appear significantly earlier, which is essential for the mechanical strength of a healing laparotomy wound.[5] Höer et al. even showed that abdominal wall sutures in rats that were gaping up to 4.8 mm showed no fascial dehiscence after 28 days and did not fail even after a pneumoperitoneum was induced.[16]

Drop-in tissue perfusion occurs within the first 4 hours after abdominal wall closure even if the wound is closed with moderate tension, most likely due to wound edema.[14] In conclusion, tension sufficient to adapt both wound edges should be used to ensure proper wound healing.

Thus, the SL:WL ratio mainly depends on the stitch length (the length of one whole stitch, measured from the near wound edge to the far edge and then back to the near edge), a parameter mainly affected by the size of the tissue bite but also by the stitch interval.[11] A short stitch length results in a stronger wound 4 days after surgery, reducing the risk of incisional hernia and wound infection and improving wound healing compared to that achieved with longer stitches.[11, 17, 18] Furthermore, smaller tissue bites reduce the amount of necrotic tissue in the wound by minimizing the amount of soft tissue that is incorporated in the suture.[1, 11, 16, 19]

A small stitch interval reduces the pressure on the tissue exerted by the suture by distributing the tension of the suture more evenly throughout the length of the incision.[17, 20] This results in a significantly higher tensile strength (almost 50% higher compared to that achieved with longer stiches).[16, 20]

Although current textbooks recommend using tissue bites 4 to 10 mm wide and stitch intervals 5 to 10 mm long for small animal abdominal wall closures, depending on the animal size,[3, 4, 21, 22] no clinical or experimental studies can be found that support these recommendations. In contrast, clinical and experimental studies suggest that a human abdomen should be closed with stitches placed 5–8 mm from the wound edge with a stitch interval of less than 5 mm.[17, 19, 20, 23]

Although many human studies on SL:WL ratios were conducted on animal models (such as pigs, rodents and rabbits), we are aware of only one study on equine cadavers dealing with the SL:WL ratio in veterinary medicine.[24, 25] In this study, the bursting pressure and failure modes of ventral median abdominal incisions closed with loop sutures in a simple continuous pattern using two different suture bite intervals were compared.

This study aimed to investigate the SL:WL ratio in an in vitro model of abdominal wall closure. How the surgeon’s level of experience affected the suturing technique and how the surgeon’s techniques changed after being educated on the SL:WL ratio were assessed. Furthermore, differences among sutures applied using SL:WL ratios below and above 4:1 using the current textbook recommendations were evaluated.

We hypothesized that small animal surgeons would apply SL:WL ratios of less than 4:1 when using their normal suturing technique and that educating the surgeons on the SL:WL ratio would significantly improve their SL:WL ratios.

## Material and methods

Three groups of ten participants each were assembled: group A (European College of Veterinary Surgery and European College of Animal Reproduction diplomates with more than 5 years of experience in small animal soft tissue surgery), group B (junior veterinary staff surgeons and residents in small animal veterinary surgery) and group C (small animal interns of veterinary medicine and surgery).

A verbal consent was obtained from each participant of the study. Each was offered to voluntarily participate or decline participation in this study, however, no specific details about why they were asked to perform sutures were discussed as to not influence the participants and their suturing behavior. A number was randomly assigned to each participant of each group and statistical analysis was performed anonymously without identification of the participants. An official ethical approval was not obtained as the authors were not aware of this requirement by the time this study was conducted.

**Fig 1:**
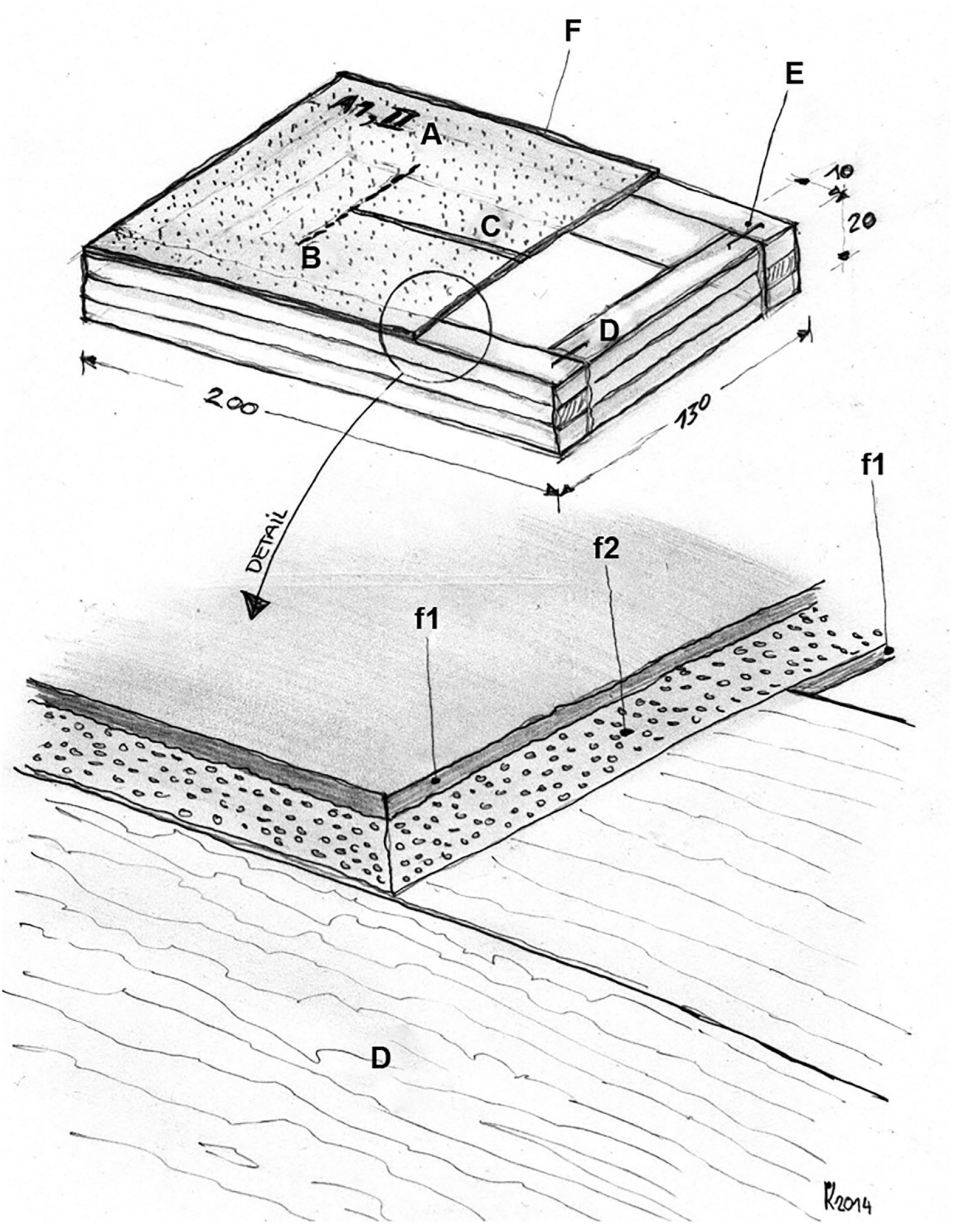
Perspective view of the suture dummy. The right part of the top region is removed to reveal the wooden frame underneath. A. Detailed view showing the layers of the artificial fascia. a. Codes for the group, number of participants and number of sutures. B. Transverse markings. C. Incision. D. Wooden frame. E. Metal staples. F. Artificial fascia with latex coating (f1) and cellular rubber (f2).

To simulate the abdominal wall, suture dummies were built by covering wooden frames (20x13 cm) with cellular rubber (Zellgummiplatten EPDM Schwarz, 2 mm, Zrunek Gummiwaren Gesellschaft m.b.H., Vienna, Austria), which was then coated with latex (Kiwoplast—Flüssiger Gummi, Kalthärtend—Hochelastisch, Kurt Wolf & CO KG, Vienna, Austria) for reinforcement. The latex surface was dusted with talcum powder to ensure easy and smooth penetration of the needle. No artificial muscle, fat, epidermal or dermal tissues were added to the dummies to simplify the evaluation of the sutures (Fig 1).

**Fig 2:**
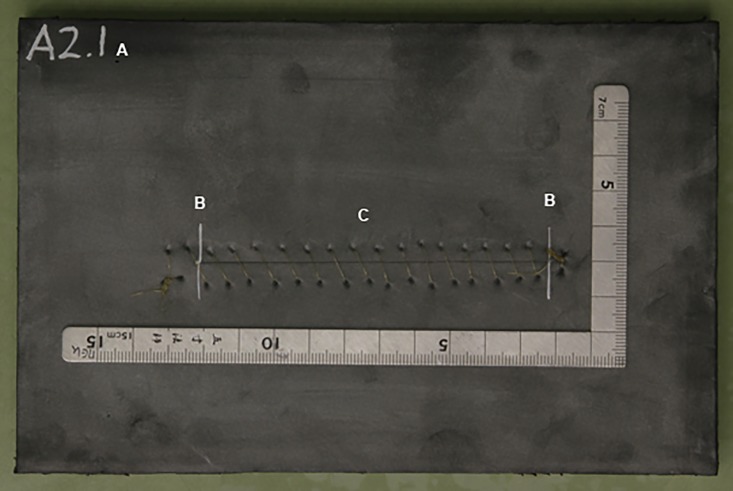
Top view of a suture dummy. a. Codes for the group, number of participants and number of sutures. B. Transverse markings. C. Sutured incision.

The dummies were marked with a three-digit code (group, number of participants and number of sutures). Two transverse markings were made 10 cm apart to mark the incision length (Fig 2).

A set of instruments consisting of Adson-Brown forceps, a Mayo-Hegar needle holder, a Mathieu needle holder and a pair of Mayo scissors was provided. Biosyn 2–0 (Biosyn, Monofilament Absorbable Suture, Covidien Austria GmbH, Brunn am Gebirge, Austria) was used for all sutures. Prior to testing, each participant received the same oral introduction: First, 3 sutures were to be performed, each one on a new suture dummy. The participants were asked to suture in the same manner as that used to close an abdominal fascia on a medium sized dog. A continuous suture pattern was required, and the knots were required to be placed outside of the 2 transverse markings. For each closure, use of a new suture material package was required. The suture dummies where incised right before the suturing. Each participant was given 60 minutes to perform 4 sutures. The first 3 closures were referred to as control sutures. Each participant was then educated on the SL:WL ratio principle and asked to attempt achieving an SL:WL ratio of 4:1 or higher on the 4th suture. For assistance, the incision length (10 cm) and total length of the suture material (75 cm) were disclosed, but no other information regarding how to achieve an SL:WL ratio of 4:1 or higher was provided. The 4th closure was referred to as the test suture.

The sutures were evaluated immediately after suturing. Each dummy was photographed with a scale for documentation. (Camera: Canon EOS 550D, Canon Inc., Tokyo, Japan; Sigma Compact Hyperzoom 28–200 mm 1:3.5–5.6, Sigma Corporation, Kawasaki, Japan; Tripod: Velbon Vel-flo 7 tripod, Velbon Tripod Co.,Ltd., Tokyo, Japan; Scale: Shinwa steel square 17x7.5 cm, Shinwa Co., Ltd., Nagoya, Japan).

The following parameters were documented:

- **Direction of suturing and use of equipment**–right to left or left to right, type of needle holder
- **Gaping**–minor gaping (0.5–1 mm), medium gaping (1–3 mm), and severe gaping (> 3 mm) were measured by applying minimal lateral tension to the sutures
- **Number of stitches–**within the 10 cm incision line
- **Suture length**–suture material was cut on both ends above the transverse markings and removed from the suture dummy and the effective length of suture material without the knot was then measured in 1 mm increments
- **Stitch interval and tissue bites–**measured for each stitch for far and near wound edges with an accuracy of 0.1 mm
- **SL:WL ratio–**calculated by dividing the effective length of suture material by the incision length (10 cm)
- **Stitch interval to tissue bite ratio–**calculated by dividing the suture’s mean stitch interval by its mean tissue bite

Suture tension was not evaluated specifically, as no viable method to asses suture tension could be found under the study’s settings.

All sutures that gaped more than 1 mm were excluded from statistical analysis (18 of 120 sutures). All sutures showing stitch intervals of 5 to 10 mm and tissue bites of 4 to 10 mm were said to follow the current textbook recommendations. Statistical Package for the Social Sciences (Statistical Package for the Social Sciences, IBM SPSS Statistics, IBM Corporation, Armonk, United States) software was used for all statistical analyses. Descriptive statistics were created for all groups combined as well as for each group separately for control and test sutures as well as for sutures below and above the SL:WL ratio of 4:1. Furthermore, descriptive statistics were created for all sutures fulfilling the current textbook recommendations for abdominal wall closure in small animals. Significant differences between the groups as well as those within the groups were tested with variance analysis and t-tests. U-tests were used if the parameters were not normally distributed. Assumptions of normal distribution were tested using the Kolmogorov-Smirnov test.

Significant differences between the combined groups for control and test sutures as well as for sutures above and below the SL:WL ratio of 4:1 were assessed with t-tests.

Correlations among the suturing parameters, the SL:WL ratio and the stitch interval to tissue bite ratio as well as correlations between the SL:WL ratio and suturing parameters were calculated using Pearson’s correlation coefficients.

For all statistical analyses, p-values below 0.05 were deemed significant.

## Results

In total, 120 sutures were analyzed, and 8,747 measurements were taken.

Of the 30 participants, 50% sutured from left to right, and 50% sutured from right to left. Only two of the 30 participants chose to use the Mathieu needle holder.

### Gaping

Forty of the 120 sutures showed gaping; 63.3% showed minor gaping, 22.4% showed medium gaping, and 14.3% showed severe gaping. Medium and severe gaping occurred independently from the SL:WL ratios above or below 4:1 regardless of whether the participant was educated on the SL:WL ratio and independently from the group. No significant correlations were found.

### Suture technique and response to education on the SL:WL ratio

No significant differences in the suturing techniques or SL:WL ratios between the individual groups or between the control and test sutures were observed (Table 1).

**Table 1:** Mean ± SD (range) suture parameters for control and test sutures.

	Combined Groups	Group A	Group B	Group C
Number of stitches—control sutures	18.2 ± 4.66	19.7 ± 5.04 (12–29)	17.0 ± 3.90 (9–28)	17.8 ± 4.73 (11–29)
Number of stitches—test sutures	20.2 ± 7.76	18.4 ± 21.2 (10–43)	18.4 ± 4.24 (13–27)	20.9 ± 6.41 (14–32)
Stitch interval (mm)—control sutures	5.43 ± 1.49	5.07 ± 1.47 (3.26–8.08)	5.72 ± 1.50 (3.31–10.94)	5.52 ± 1.47 (3.09–8.87)
Stitch interval (mm)—test sutures	5.16 ± 1.78	5.38 ± 2.58 (2.17–9.50)	5.28 ± 1.11 (3.58–7.31)	4.83 ± 1.40 (2.89–6.74)
Tissue bites (mm)—control sutures	5.25 ± 1.47	5.02 ± 1.47 (3.45–8.93)	5.93 ± 1.55 (3.39–8.82)	4.75 ± 1.10 (2.84–7.82)
Tissue bites (mm)—test sutures	5.99 ± 2.13	6.42 ± 2.65 (3.61–10.55)	6.64 ± 2.09 (4.12–9.83)	4.97 ± 1.24 (3.54–6.93)
SL:WL ratio—control sutures	4.14 ± 1.08	4.14 ± 0.91 (2.45–5.48)	4.44 ± 1.02 (2.10–6.40)	3.81 ± 1.26 (1.54–6.74)
SL:WL ratio—test sutures	4.87* ± 0.97	5.08* ± 0.85 (3.52–6.12)	5.06 ± 0.63 (3.85–5.98)	4.50 ± 1.28 (2.91–6.81)

*Value differs significantly (P < 0.05) from the value of the control sutures.

60.5% of the control sutures and 77% of the test sutures showed SL:WL ratios higher than 4:1 (see Table 2 for the individual group results). Even though no significant correlation was observed between surgeons educated on the SL:WL ratio and ratios above 4:1 (p = 0.131), a statistically significant increase in the ratio was observed in the test sutures when assessing the SL:WL ratios of all the groups combined (Table 1). For the individual groups, only group A showed a significant difference in the SL:WL ratio before and after education on its principles (p = 0.003). Furthermore, only groups A and B achieved mean SL:WL ratios above 4:1 in the control sutures. In the test sutures, all 3 groups achieved mean ratios above 4:1.

**Table 2:** Total number of sutures and number and percentage of sutures above SL:WL ratio 4:1.

	Total	>4:1	%
Group A—control sutures	27	17	63.0%
Group B—test sutures	9	8	88.9%
Group B—control sutures	26	19	73.1%
Group B—test sutures	8	7	87.5%
Group B—control sutures	23	10	43.5%
Group C—test sutures	9	5	55.5%

When trying to achieve an SL:WL ratio of 4:1 or higher, group A participants increased their mean tissue bite size by 1.4 mm and their mean stitch interval by 0.31 mm. Group B participants increased their tissue bite size by 0.71 mm but decreased their stitch interval by 0.44 mm, while group C participants increased their tissue bite size by 0.22 mm but decreased their stitch interval by 0.69 mm.

Altogether, the surgeons tended to decrease their stitch interval, thus increasing the number of stitches and the size of the tissue bites.

### SL:WL ratios below and above 4:1

**Fig 3:**
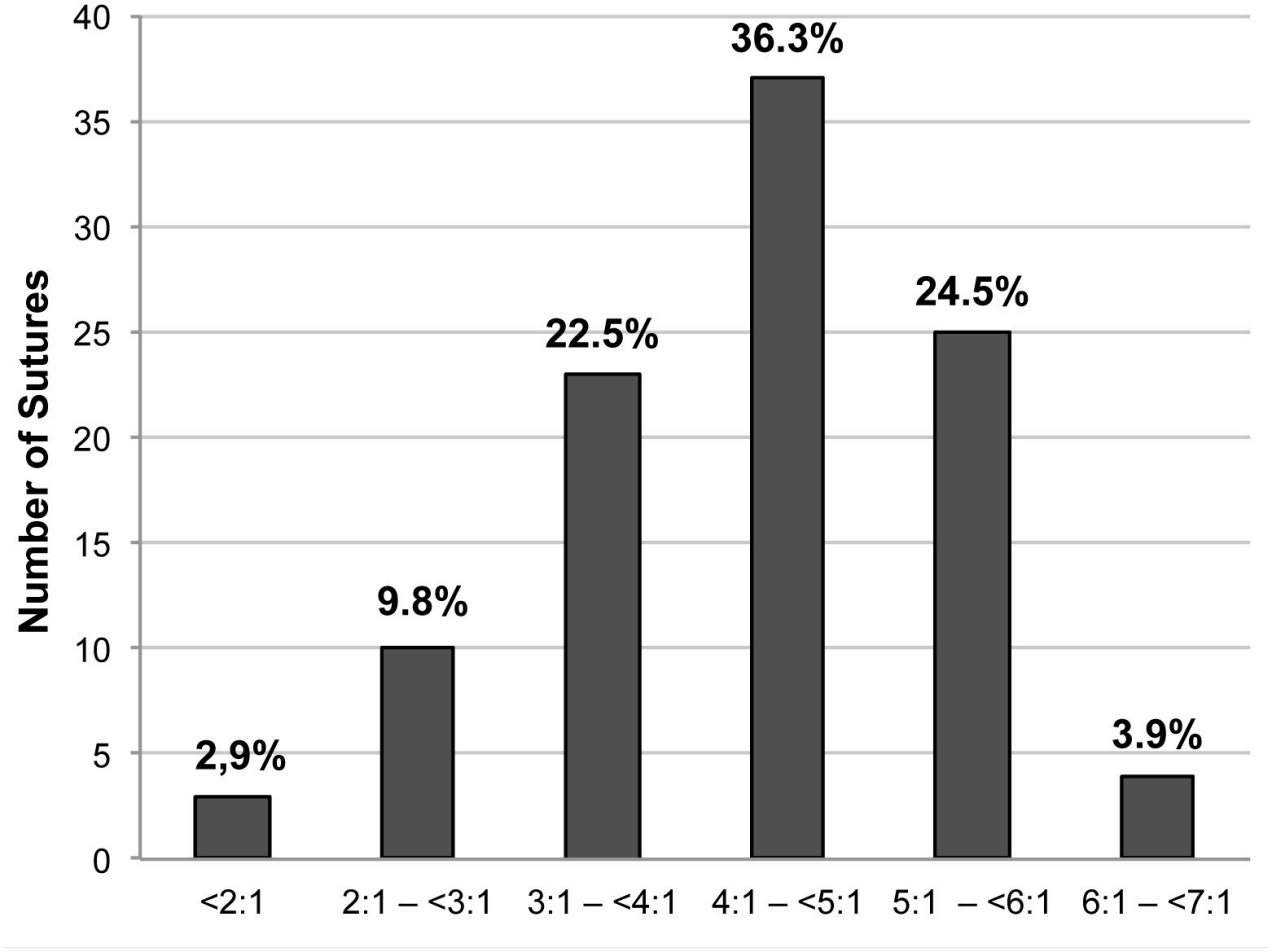
Graphical illustration of the quantitative distribution of segregated SL:WL ratios.

Overall the SL:WL ratios ranged from 1.54:1 to 6.81:1, with most sutures lying between 4:1 and 5:1 (36.3%). Only three sutures had a ratio below 2:1 (Fig 3). Sutures above a SL:WL ratio of 4:1 showed significantly higher stitch intervals (p < 0.001), significantly larger tissue bites (p = 0.003) and significantly higher numbers of stitches (p < 0.001) compared to sutures below 4:1 (Table 3). No significant differences in suturing technique for sutures above and below the SL:WL ratio of 4:1 were observed among the three groups.

**Table 3:** Mean ± SD (range) suture parameters for sutures with SL:WL ratio <4:1 and ≥4:1.

	Number of stitches	Stitch interval	Tissue bites	SL:WL ratio
Sutures with SL:WL ratio <4:1	16.2 ± 3.8	6.08 ± 1.56	4.71 ± 0.98	3.13 ± 0.63
	(9–26)	(3.87–10.94)	(2.84–7.77)	(1.54–3.95)
Sutures with SL:WL ratio ≥4:1	20.11* ± 6.0	4.96* ± 1.42	5.83* ± 1.85	4.98* ± 0.68
	(11–43	(2.17–8.42)	(3.27–10.55)	(4.01–6.81)

*Value differs significantly (P < 0.05) from the value of sutures with SL:WL ratio <4:1.

Sutures between 4:1 and 5:1 had a mean stitch interval of 5.17 ± 1.47 mm (3.09–8.42 mm) and a mean tissue bite size of 5.52 ± 1.75 mm (3.27–10.55 mm).

**Fig 4:**
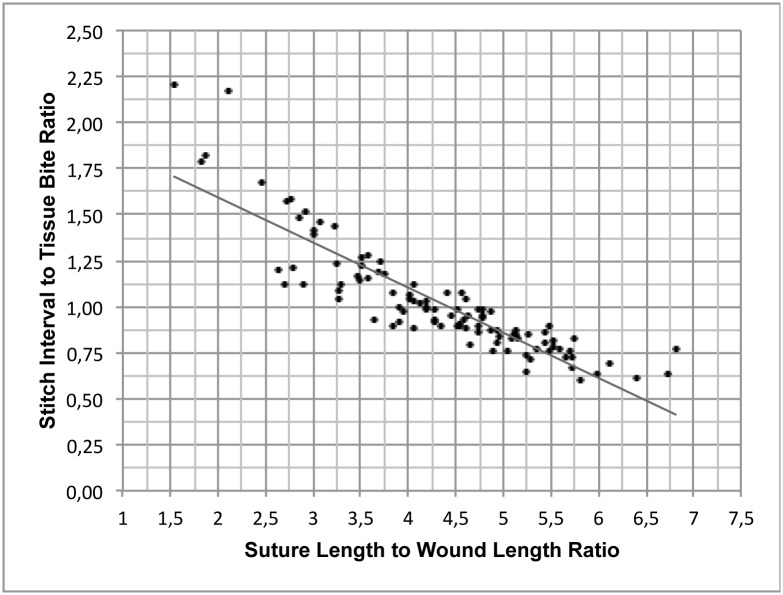
Ratio of mean stitch intervals to tissue bites plotted against the corresponding SL:WL suture ratio.

A significant negative correlation (rs = -0.886) was found between the stitch interval to tissue bite ratio and the SL:WL ratio (p < 0.001) (Fig 4), which ranged from 1.46:1 (SL:LW ratios between 2:1–3:1) to 0.67:1 (SL:WL ratios between 6:1–7:1). For sutures with SL:WL ratios between 4:1 and 5:1, the stitch interval to tissue bite ratio was 0.95:1 (Table 4).

**Table 4:** Stitch intervals and tissue bites (mm) and corresponding stitch interval to tissue bite ratio for segregated SL:WL ratio.

SL:WL ratio		mean	SD	range	Stitch interval to tissue bite Ratio
2:1 –<3:1	Stitch interval	6,06	1,97	3.87–10.94	1.46:1
	Tissue bite	4,08	0,48	3.45–5.04	
3:1 –<4:1	Stitch interval	5,95	1,33	3.96–9.50	1.17:1
	Tissue bite	5,11	0,94	3.40–7.77	
4:1 –<5:1	Stitch interval	5,17	1,47	3.09–8.42	0.95:1
	Tissue bite	5,52	1,75	3.27–10.55	
5:1 –<6:1	Stitch interval	4,8	1,38	2.17–7.54	0.77:1
	Tissue bite	6,26	2	3.54–10.49	
6:1 –<7:1	Stitch interval	4,02	0,87	2.89–3.75	0.67:1
	Tissue bite	6,09	1,7	4.91–7.82	

### Sutures fulfilling the textbook recommendations

In total, 49 sutures (48.0%) fulfilled the current textbook recommendations. The mean SL:WL ratio was 4.10:1 (range 1.54:1–5.98:1), but only 27 (26.5%) sutures showed ratios above 4:1 (6 in group A, in group B and 5 in group C).

### Correlation analysis of suture parameters

A high negative correlation was found between the number of stitches and the stitch interval (rs = -0.986). Medium correlations were found between tissue bite size and the number of stitches (rs = -0.561) and the stitch interval (rs = 0.573). Finally, small correlations were found between the SL:WL ratio and the number of stitches (rs = 0.391), the stitch interval (rs = -0.396) and the tissue bite size (rs = 0.383).

## Discussion

To our knowledge,[25] this is the first study to assess the surgical techniques of small animal surgeons performing abdominal wall closures and evaluating their SL:WL ratios using an in vitro model. We explored how the surgeon’s level of experience affected the suturing technique and how the surgeon’s techniques changed after being educated on the SL:WL ratio.

The hypothesis that small animal surgeons would not apply an SL:WL ratio of 4:1 or higher with their normal suturing technique was not proven. However, educating surgeons on the SL:WL ratio did significantly improve their ratios. In total, 60.5% of the control sutures showed an SL:WL ratio above 4:1, demonstrating that most surgeons achieved this ratio using their normal suturing behavior. No significant differences in the SL:WL ratio could be found between the groups. However, as nearly 70% of the control sutures made by groups A and B had ratios above 4:1 and almost 90% of their test sutures met this ratio, a trend that more experienced surgeons were more likely to achieve a ratio higher than 4:1 than novices was observed. Additionally, group A participants achieved the highest improvement in the SL:WL ratio after being educated on its principles, suggesting that more experienced surgeons can better utilize the information and adapt their suturing behavior.

No significant differences regarding the sutures parameters were observed between the groups nor between the control and test sutures. This result could be due to the small group sizes and the wide variation within the groups. Even in the group of diplomates, who all had excellent surgical experience, wide ranges of stitch intervals and tissue bite sizes were observed. Thus, suturing techniques appear to be influenced by personal preference and the surgeons’ habits.

When sutures with SL:WL ratios above 4:1 were compared to those with ratios below 4:1, significant differences were observed in the tissue bite size and, even more dramatically, in the stitch interval (and thus also in the number of stitches). This shows that the main difference between sutures with high and low ratios is the stitch interval.

Additionally, we found a moderate correlation between the stitch intervals and tissue bite size.

High SL:WL ratios are therefore most likely achieved in 2 ways: using small stitch intervals in combination with small tissue bites (resulting in a small stitch length overall) or using large tissue bites with large stitch intervals (resulting in a large stitch length overall). This finding resembles those reported by Cengiz^23^ and Milbourn^10^.

Sutures fulfilling the current textbook recommendations for abdominal wall closure (5–10 mm stitch interval, 4–10 mm tissue bites) showed a mean SL:WL ratio of 4.1:1, showing that achieving this ratio following those recommendations is possible. However, these sutures also showed an extremely wide range of SL:WL ratios (1.54:1–5.98:1). With the average patient in small animal surgery being smaller than a human, using wider tissue bites and stitch intervals than those suggested for human subjects seems unnecessary.

Together, the results of this study suggest that stitch intervals from 4 to 5.5 mm and tissue bites from 4.5 to 6 mm should give a reasonable range, which should result in an SL:WL ratio between 4:1 and 5:1 if followed.

In this study, a relationship between the stitch interval to tissue bite ratio and the SL:WL ratio was elucidated, which was a major finding. For sutures with low SL:WL ratios, the stitch interval was shown to be bigger than the tissue bites, and in sutures with higher SL:WL ratios, the stitch interval gradually became smaller than the tissue bites. For SL:WL ratios between 4:1 and 5:1, the mean stitch interval was 95% of the mean tissue bite size. Due to its high significant correlation with the SL:WL ratio, the stitch interval to tissue bite ratio might be a feasible method for predicting suture SL:WL ratios in a clinical setting. This would provide easy and reliable guidance for acquiring a desirable SL:WL ratio in any suture.

Notably, this study also has a few limitations. First, the tests were performed on an in vitro model. Even though the homemade suture dummies were sufficient, they might not sufficiently compete with commercially available suture dummies. Furthermore, an artificial model can never reproduce real conditions. Israelsson^13^ showed that even introducing new suture material in a clinical setting altered the suturing behavior of surgeons, indicating that surgeons might achieve different SL:WL ratios when performing abdominal wall closures on real patients. In the literature, suture parameters are usually obtained by counting the number of stitches after the suture is finished and measuring the length of the closed incision. The suture length, mean stitch length and mean stitch interval can then be obtained mathematically. In this study, artificial models were used to create the same conditions for all surgeons. Furthermore the model made it possible to remove the thread from an exact incision length and to separately measure each stitch interval and tissue bite for the far and near wound edges without worrying distortion were possible.

Another limitation was the small group size. Ten surgeons per group is sufficient for providing a decent overview for surgeons having different experience levels, but the results must be interpreted with caution due to high within-groups variations in combination with the small group size.

## Conclusions

By following the current textbook recommendations and applying a stitch interval close to the tissue bite size, an SL:WL ratio equal to or larger than 4:1 can be achieved using an in vitro laparotomy closure model.

## Supporting information

S1 File Raw data. (XLS)
